# Seasonal Dynamics Are the Major Driver of Microbial Diversity and Composition in Intensive Freshwater Aquaculture

**DOI:** 10.3389/fmicb.2021.679743

**Published:** 2021-06-24

**Authors:** Sophi Marmen, Eduard Fadeev, Ashraf Al Ashhab, Ayana Benet-Perelberg, Alon Naor, Hemant J. Patil, Eddie Cytryn, Yehudit Viner-Mozzini, Assaf Sukenik, Maya Lalzar, Daniel Sher

**Affiliations:** ^1^Department of Marine Biology, Leon H. Charney School of Marine Sciences, University of Haifa, Haifa, Israel; ^2^Department of Functional and Evolutionary Ecology, University of Vienna, Vienna, Austria; ^3^Microbial Metagenomics Division, The Dead Sea and Arava Science Center, Masada, Israel; ^4^Dor Aquaculture Research Station, Fisheries Department, Israel Ministry of Agriculture and Rural Development, Dor, Israel; ^5^Institute of Soil, Water and Environmental Sciences, Volcani Center, Agricultural Research Organization, Rishon Lezion, Israel; ^6^The Yigal Allon Kinneret Limnological Laboratory, Israel Oceanographic and Limnological Research, Migdal, Israel; ^7^Bioinformatics Service Unit, University of Haifa, Haifa, Israel

**Keywords:** microbiome, fishpond, 16S rRNA, cyanobacteria, runoff

## Abstract

Aquaculture facilities such as fishponds are one of the most anthropogenically impacted freshwater ecosystems. The high fish biomass reared in aquaculture is associated with an intensive input into the water of fish-feed and fish excrements. This nutrients load may affect the microbial community in the water, which in turn can impact the fish health. To determine to what extent aquaculture practices and natural seasonal cycles affect the microbial populations, we characterized the microbiome of an inter-connected aquaculture system at monthly resolution, over 3 years. The system comprised two fishponds, where fish are grown, and an operational water reservoir in which fish are not actively stocked. Clear natural seasonal cycles of temperature and inorganic nutrients concentration, as well as recurring cyanobacterial blooms during summer, were observed in both the fishponds and the reservoir. The structure of the aquatic bacterial communities in the system, characterized using 16S rRNA sequencing, was explained primarily by the natural seasonality, whereas aquaculture-related parameters had only a minor explanatory power. However, the cyanobacterial blooms were characterized by different cyanobacterial clades dominating at each fishpond, possibly in response to distinct nitrogen and phosphate ratios. In turn, nutrient ratios may have been affected by the magnitude of fish feed input. Taken together, our results show that, even in strongly anthropogenically impacted aquatic ecosystems, the structure of bacterial communities is mainly driven by the natural seasonality, with more subtle effects of aquaculture-related factors.

## Introduction

Freshwater environments come in many shapes and forms, from pristine mountain lakes to highly polluted wastewater treatment facilities, each hosting a complex and dynamic microbial community (“microbiome”; Bruno et al., 2018; Bautista-de los Santos et al., 2019; Zorz et al., 2019). Such aquatic microbiomes are strongly affected by environmental conditions within the water body (e.g., temperature and nutrients availability), and in turn shape its biogeochemistry, for example by processing and recycling organic and inorganic matter (Liu et al., 2017; Rowe et al., 2018; Hach et al., 2020). Aquatic microbial communities may also affect other organisms living within the water body, as well as human communities in contact with the water. For example, the eggs and larvae of some aquatic organisms are inoculated by microbes from the surrounding water, in turn affecting the health and fitness of these organisms (Grześkowiak et al., 2012; Pérez-Sánchez et al., 2014; Song et al., 2014; Giatsis et al., 2016). Similarly, aquatic microbiomes may serve as pools of various pathogens, and some aquatic microorganisms (primarily prokaryotic and eukaryotic microalgae) can produce toxins (Paerl et al., 2018; Wurtsbaugh et al., 2019). Given the importance of freshwater microbiomes to water quality and environmental health worldwide, significant effort has been invested in understanding the interplay between environmental conditions and microbiome structure and function (reviewed by Orland et al., 2019).

Freshwater aquaculture facilities such as fishponds are an example of an aquatic ecosystem that is significantly impacted by anthropogenic activity. The growth of human population and the concomitant increase in the demand for animal-based protein have resulted in a rapid expansion of aquaculture worldwide, a process also termed a “blue revolution” (reviewed by Garlock et al., 2020). The aquaculture industry has been growing at an average rate of ca. 6–8% since the 1970s, and now supplies more fish and seafood biomass than natural catch (Ahmed and Thompson, 2019; Garlock et al., 2020). As a result, fishponds (as an ecosystem) are becoming more abundant, and their interaction with surrounding environments is increasing.

Modern intensive aquaculture practices rely on growing large stocks (i.e., high biomass) of fish or other aquatic organisms in a limited area. This requires large amounts of fish-feed that often leads (together with fish excrement) to hyper-eutrophic conditions (Gowen, 1994; Talbot and Hole, 1994; Ahmed and Thompson, 2019). Additionally, aquaculture is often performed in monoculture (i.e., a single type of organism is grown) or in polyculture of a limited number of organisms, and the combination of ultra-dense populations and hyper-eutrophic conditions (i.e., poor water quality) can lead to disease outbreaks (Glibert et al., 2002; Mennerat et al., 2010; Ahmed and Thompson, 2019). As a result, aquaculture often supports fish health by using drugs and antibiotics, as well as algaecides in the case of algal blooms. However, despite the ubiquity of such aquaculture husbandry practices, and their potential impact on aquatic microbial populations, little is known about the temporal dynamics of the microbiome in fishponds, and how it responds to natural and anthropogenic perturbations (Rodriguez-Brito et al., 2010;Patil et al., 2020).

**FIGURE 1:**
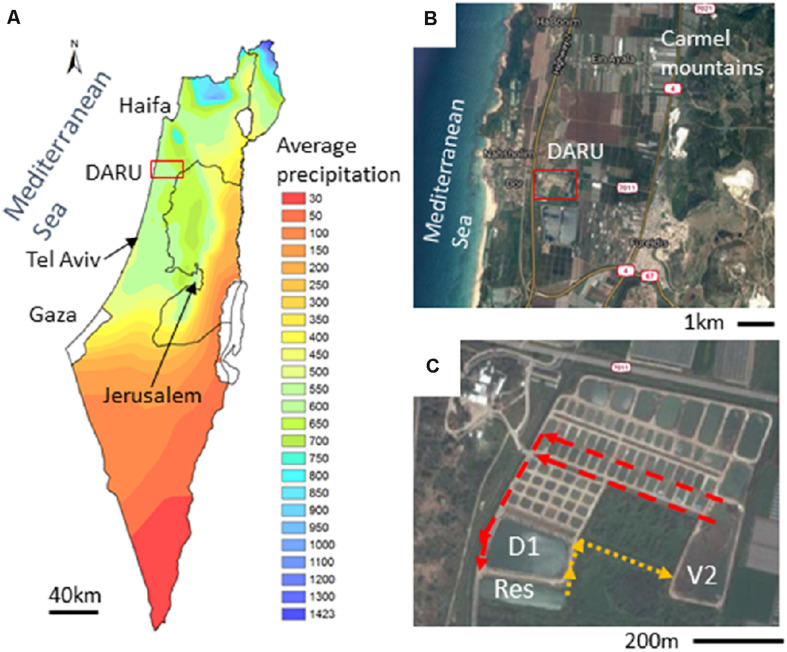
The study site - Dor Aquaculture Research Unit (DARU). **(A)** Mean annual precipitation across Israel with a characteristic Mediterranean climate in the coastal regions. **(B)** The coastal plain between the Carmel mountains and the Mediterranean Sea. The red square in panels **(A)** and **(B)** marks the location of DARU. **(C)** The fishponds infrastructure at DARU. The arrows show the flow direction between the D1 and V2 fishponds and the Reservoir. The satelite images were acquired from Google maps. The mean annual precipitation map of Israel was derived from the digital elevation model (DEM) using ArcGIS (ESRI, Redlands, CA, United States).

To address these questions, we characterized, over 3 years, temporal microbiome dynamics in two fishponds connected to a semi-natural operational water reservoir, which recycles the fishpond water (Figure 1, see detailed description below). Our aim was to identify factors that dictate the water microbiome composition of these highly anthropogenically impacted ecosystems. These included the quality of the water, phytoplankton populations, as well as to the types of fish present and their feeding regimen. We hypothesized that specific intensive fishery practices in each fishpond (e.g., the types of fish raised and their feeding regime) would shape the microbiomes of the fishponds, despite the connectivity between them. This would result in different populations between the fishponds, as well as between them and the reservoir. Our results, however, point to seasonality rather than specific aquaculture practices as the major force driving the microbial population structure.

## Materials and Methods

### Description of the Study Site

The Dor Aquaculture Research Unit (DARU - 32°36′25.4′′N 34°55′54.7′′E) is situated on the coastal plain of Israel (Figure 1). DARU belongs to the Ministry of Agriculture and Rural development of Israel and operates as a research unit and a semi-commercial outgrowth facility. The research at DARU is performed in approximately 80 miniature ponds (0.03 hectare), and focuses on genetically-informed breeding programs, increasing fish health by testing new immunization methods and developing healthier and more ecologically-sound methods of intensive fish rearing (Patil et al., 2016). In addition, DARU has two larger ponds, termed D1 and V2 (1.8 and 1.6 hectares each, with 18,000 and 12,000 m^3^ of water, respectively), which are used for semi-commercial outgrowth of Common carp (*Cyprinus carpio*) and Silver carp (*Hypophthalmichthys molitrix*). All the ponds are connected to an external, one-hectare, operational reservoir (ca. 8,000 m^3^), which is used to regulate the water levels and temperature in the fishponds. Water is supplied to the reservoir from a local well and from floodwater during the winter months. The ponds and the reservoir are connected by a series of channels, where plants are maintained with the aim of providing “biofiltration” (Figure 1).

During the 3-year study period, fishpond V2 was stocked with Common carp, whereas fishpond D1 was stocked with Silver carp in 2013 and with Common carp in 2014–2015 (Supplementary Table 1). The outgrowing period of the Common carp is typically about 5–7 months whereas that of a Silver carp is shorter (ca. 2–3 months), both occurring during the spring and summer months (i.e., March–September). When no fish are grown the fishponds are kept dry, with the exception of occasional flood-water puddles at the deepest part of the fishpond. At the initiation of the outgrowth period, two-thirds of the fishponds’ volume is filled with water pumped from the operational reservoir, and one-third with fresh groundwater from a local well. During the growing period, the water temperature is regulated by adding cold water from the well, or warmer water from the reservoir. At the end of the growing period, water from the fishponds is transferred through connecting channels to the operational reservoir, and then via a natural stream to the Mediterranean Sea. The reservoir contains water throughout the entire year, and was sampled almost monthly, and primarily during the outgrowth period in fishponds D1 and V2.

### Sampling and Analysis of Environmental Data

Monthly samples were collected at the reservoir and fishponds D1 and V2 (when they contained water) from 2013 to 2015 (Supplementary Table 1). In some cases, samples were collected outside of the active stocking period, when no or few fish were present. Each pond was sampled at a constant location from the edge of the water body and included measurement of dissolved oxygen, temperature and pH, using field probes (Eutech instruments, Singapore). At each location, five liters of surface water were collected using plastic jerrycans (specific to each location) that were washed with double distilled water and with 70% ethanol prior to sampling. Within 1 h the samples were transferred in darkness to the lab and processed according to the methodology described below.

For measurement of pigments, water was filtered through GF/C filters (glass fiber filter, 13 mm, nominal pore size 1.2 μm; Whatman plc, Maidstone, United Kingdom) and placed into 1.5 mL sterile Eppendorf tubes. The water was filtered until the filters were clogged, and the volume was recorded (30–150 mL). For DNA extraction and microcystin measurements, water was filtered through GF/F filters (glass fiber filter, 13 mm, nominal pore size 0.7 μm; Whatman plc, Maidstone, United Kingdom). The filters were placed into 1.5 mL sterile tubes, and for the purpose of DNA extraction were covered with 1 mL of storage buffer (40 mM EDTA, 50 mM Tris-HCl, 0.75 M Sucrose) and stored at −80°C until analyzed. The filtrate from the GF/F filters was collected for measurement of dissolved inorganic nutrients, and were maintained at −20°C until analysis.

### Inorganic Nutrients Measurements

The concentrations measurements of PO_4_^+^, NH_4_^+^, NO_3_^–^, and NO_2_^–^ in fishponds and in the water reservoir were measured using the AA3 Segmented Flow Multi-Chemistry Analyzer (SEAL Analytical, Germany), following the manufacturer protocols (Ammonia: Method no. G-327-05 Rev. 7 - Fluorescent method; Nitrate and Nitrite: Method no. G-172-96 Rev. 17 and Phosphate: Method no. G-297-03 Rev. 5). Inorganic nutrients in the rainwater runoff were measured using a Lachat Autoanalyzer (Lachat Instruments, Milwaukee, WI, United States) by the manufacturer’s protocol. For calculations of the N:P ratio, we present the ratio of the sum of NH_4_^+^, NO_3_^–^ and NO_2_^–^ to PO_4_^+^, noting that other measurements of the limiting elements may lead to different interpretations of the data (Kosten et al., 2009).

### Photosynthetic Pigment Analysis

Pigments were extracted for 3 h in absolute methanol in the dark. Pigment extractions were immediately clarified with syringe filters (Acrodisc CR, 13 mm, 0.2 μm PTFE membranes; Pall Life Sciences, New York, NY, United States) and transferred to glass ultra-performance liquid chromatography (UPLC) vials. The samples were preheated to 30°C, and 10 μl were injected into an ACQUITY UPLC system (Waters Corporation, Milford, MA, United States) equipped with a C8 column (1.7 μm particle size, 2.1 mm internal diameter, 50 mm column length, ACQUITY UPLC BEH) heated to 50°C and a guard column. The protocol was based on the LOV method (Hooker and McClain, 2000), with some modifications: the mobile phase gradient consisted of a mixture of 70:30 methanol: 0.5 M ammonium acetate as solvent A and 100% methanol as solvent B. Peaks were monitored at 440 nm and their absorbance spectra was determined using a photodiode array (PDA) detector. Known standards of Chlorophyll a, Chlorophyll b, Chlorophyll c_2_, Zeaxanthin, β-carotene, Diatoxanthin, Dinoxanthin, Fucoxanthin, and Peridinin were separated before each run for further identification and quantification. All standards were purchased from the DHI Laboratory (Hørsholm, Denmark). Pigments were identified by retention time and spectrum absorbance (obtained by PDA detector reads at 350–700 nm). Pigment concentrations were adjusted to 1 mL extraction volume and filtration volume. While zeaxanthin is found in multiple organisms, it is associated primarily with cyanobacteria and, as phycobiliproteins are not measurable with this chromatography method, were considered a diagnostic pigment for these organisms (Wright et al., 2005).

### Fish Biomass and Supplied Food Mass Estimation

Fish biomass and the fish-feed input were estimated for the sampling time-points based on the limited records kept at the Dor Aquaculture Research Unit (DARU). When the fishponds were established for the growing season, the total number and average biomass of the larvae or juvenile fish was recorded. Once or twice a month, during the outgrowth period, a representative sample of fish was weighed and returned to the water. The number of fish in the water was estimated and the amount of fish-feed supplied was recorded. Based on these measurements, we calculated the fish biomass and used a fitted regression model (function ‘lm’ in R package ‘stats’ v4.0.0) to estimate the fish biomass when our samples were taken, which was always during the outgrowth period (Supplementary Figure 2). A similar approach was applied to estimate the amount of food added to the fishponds (Supplementary Figure 2). We would like to emphasize that these models are not designed to be predictive or mechanistic and are limited to interpolating the data to provide estimates of the biomass and feed mass during the sampling times.

### Microcystin Extraction and Measurement

**FIGURE 2:**
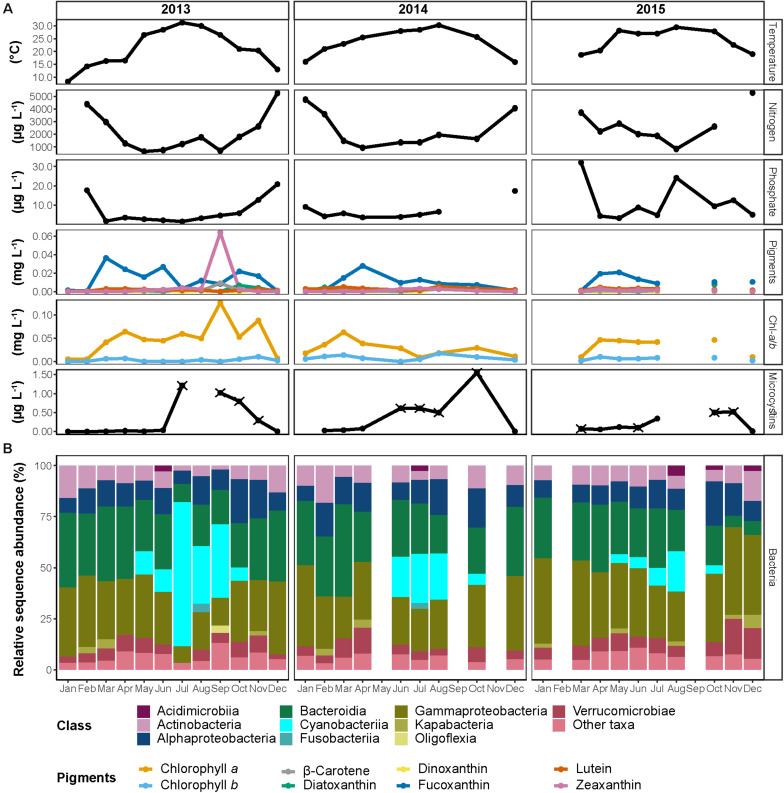
Seasonal dynamics in the Reservoir between 2013 and 2015. **(A)** Monthly measured physicochemical properties of the water. The different photosynthetic pigments are colored according to the legend. Nitrogen represents the total concentration of nitrate and nitrite. In microcystins, samples marked with ‘×’ represent potentially underestimated concentration. For visualization purposes, microcystins measurements of October 2014 and August 2015 (1.6 and 10.4 μg L^–1^, respectively) were omitted from the figure. **(B)** Sequence proportion overview of bacterial communities on a class level. The classes represented by colors according to the legend, all classes with sequence proportions below 2% were classified as “Other classes.”

Cyanotoxins (microcystins or nodularins) in phytoplankton biomass were measured immunologically. Biomass collected on filters was subjected to methanolic extraction (3 h incubation in 1 mL 100% Methanol), followed by 15 min sonication. The upper 500 μL were dried under vacuum for 2 h in a speedvac, resuspended in 50 μL of 75% methanol and further dilution to 4% methanol. A subsample of 50 μL of that solution was used for quantification by the Microcystins/Nodularins (ADDA) Elisa Kit (Eurofins Abraxis Inc., Warminster, PA, United States). This kit cannot differentiate between these toxins, and we refer to the results as microcystins throughout this study. This protocol led to a concentration factor of 5–100 (depending on the initial filtered volume), bringing the samples within the range of sensitivity of the measuring kit (0.1 μg L^–1^). In some cases, the ELISA results were above the linear range of the kit. Due to technical reasons these samples were not diluted and re-measured, and therefore, these samples (marked in Figure 2, Supplementary Figure 1, and Supplementary Table 2) should be considered as likely underestimates.

### DNA Isolation and 16S rRNA Amplicon Sequencing

The DNA extraction was performed using a combination of manual treatments and robotic extraction with the QiaCube robot (Biomedical Core Facility, Technion, Haifa, Israel). Briefly, filters were thawed, centrifuged for 10 min at 15,000 × *g* and the storage buffer was removed. Lysis buffer from the DNeasy Blood & Tissue Kit (Qiagen, Hilden, Germany) was added and the samples were mechanically pounded with two 3 mm sterile stainless steel beads at the speed of 30 Hz for 1.5 min using a TissueLyser LT (Qiagen, Hilden, Germany). After addition of 30 μl lysozyme the tubes were incubated at 37°C for 30 min, followed by the addition of 25 mL proteinase K and 200 μL of the buffer AL with an additional incubation for 1 h at 56°C on a shaker. Finally, the tubes were centrifuged for 10 min at 5,000 × *g* and the upper liquid was transferred to a new 2 mL tube for extraction by the QiaCube robot, according to manufacturer’s instructions (Qiagen, Hilden, Germany). The DNA quantity was assessed using Picogreen (Quant-iT PicoGreen dsDNA reagent; Invitrogen, Carlsbad, CA, United States), and the quality (apparent size and degradation products) was assessed using a TapeStation (Agilent Technologies, Santa Clara, CA, United States).

Polymerase chain reaction (PCR) reactions for amplifying 16S rRNA gene fragments were performed in triplicate for each DNA sample, using the following primers that target the V3–V4 region: forward CS1_341F (Muyzer et al., 1993) (5′-ACA CTG ACG ACA TGG TTC TAC ANN NNC CTA CGG GAG GCA GCA G-3′) and reverse CS3_806R (Caporaso et al., 2011) (5′-TAC GGT AGC AGA GAC TTG GTC TGG ACT ACH VGG GTW TCT AAT-3′). The PCR reactions were performed in a final volume of 25 μl, with 10 ng of DNA template. The PCR protocol was as follows: initial denaturation stage at 95°C for 5 min, followed by 28 cycles at 95°C for 30 s, 50°C for 30 s, and 72°C for 60 s, with a final extension step at 72°C for 5 min, using BIOLINE 2x MyTaq Red Mix (Sigma-Aldrich, St. Louis, MO, United States), and the PCR reaction was performed in a TProfessional Basic Gradient thermocycler (Biometra, Göttingen, Germany). Following the first PCR, triplicate samples were pooled, and the products were sent to the DNA Services Facility of the University of Illinois, Chicago, where a second PCR was performed to incorporate barcodes and sequencing adapters (CS1 and CS3). Sequencing was performed using a 2 × 250 base pair format on a MiSeq flow cell (V3 chemistry, Illumina, United States). The 2013–2014 and 2015 samples were sequenced at different times and on two separate sequencing lanes (see section “Results”). The raw sequencing reads are available as BioProject PRJNA688121 at the NCBI SRA database.

### Bioinformatics and Statistical Analyses

The raw paired-end reads were primer-trimmed using cutadapt (Martin, 2011), and subsequent analyses of the generated sequences were conducted using R (v4.0.0^1^) in RStudio (v1.2.5033^2^) as described below. The trimmed libraries were processed using DADA2 (Callahan et al., 2016) (v1.14.1), following the suggested tutorial^3^. Initially, chimeras and singletons were filtered out, and subsequently, amplicon sequence variants (ASVs) were taxonomically classified against the Silva reference database (release 138) (Quast et al., 2012). The ASVs that were taxonomically unclassified to the domain level, or not assigned to bacterial or archaeal lineages, were excluded from further analysis. Furthermore, all ASVs which were taxonomically assigned to mitochondria and chloroplast were removed from the dataset.

Sample data matrices and alpha diversity indices were managed using the R package ‘phyloseq’ (v1.28.0; McMurdie and Holmes, 2013). Plots were generated using R package ‘ggplot2’ (v3.3.0; Wickham, 2016). Calculation and visualization of shared ASVs was conducted using R package ‘venn’ (v1.9; Dusa, 2020). Kruskal–Wallis tests with a *post hoc* Wilcoxon signed-rank test, as well as Person’s correlations, were conducted using the R package ‘rstatix’ (v0.7.0; Kassambara, 2021). The sample rarefaction curves and their estimated asymptotic extrapolation were conducted using R package ‘iNEXT’ (v2.0.20; Hsieh et al., 2020). Permutational multivariate analyses of variance (PERMANOVA) were conducted using function ‘adonis2,’ and redundancy analyses (RDA) were conducted using a stepwise ordination significance test ‘ordistep,’ both functions are from the R package ‘vegan’ (v2.5.7; Oksanen et al., 2020) and were conducted with 999 permutations. Both analyses were conducted on a transformed ASV table using a geometric mean. Environmental variables that exhibited significant collinearity were excluded from the RDA analyses. The fold-change in abundance of each ASV between the water layers was calculated using the R package ‘DEseq2’ (v1.24.0; Love et al., 2014). The method applies a generalized exact binomial test on variance stabilized ASV abundance. The ASVs within each genus were defined as enriched when they had a log_2_ fold change >1 and an adjusted *p-*value < 0.1, after correction for multiple-testing according to Benjamini and Hochberg (1995).

Scripts for the molecular data processing and statistical analyses can be accessed at https://github.com/edfadeev/Dor_ponds_16S.

## Results

### Water Quality Parameters Change Seasonally in the Reservoir

We first focus on the operational reservoir, which contains water throughout the entire year, is not actively stocked with fish, and provides a baseline for the system over the 3 years of sampling (Figure 2). As expected from a water body situated in a typical Mediterranean climate (cold and wet winters and hot and dry summers), and despite the capacity at DARU to regulate water temperature to some extent by adding well-water, the temperature showed a clear seasonal cycle, ranging from 10–20°C during the rainy season (November–April; further addressed as ‘wet season’) to 20–30°C during the dry season (May–October; further addressed as ‘dry season’; Figure 2). Although some variability was observed from year to year, inorganic nitrogen (nitrate, nitrite, and ammonia combined; further addressed as ‘N’) and soluble reactive phosphate (SRP; further addressed as ‘P’) were typically low during the summer months, with minimum concentrations of 600 and 2 μg L^–1^, respectively. The concentrations began rising in autumn and reached the highest concentrations during the winter months, 5,322 and 2 μg L^–1^, respectively. Importantly, we had expected that the nutrients in the reservoir would be higher during the fish outgrowth period (spring and summer), when intensive fish feeding occurs, as a potential result of water from the fishponds reaching the reservoir (Supplementary Figure 1). The observation that nutrient concentrations peaked during winter prompted us to search for other, non-aquaculture related, processes that might have provided nutrient input to the reservoir. One such process is rainwater runoff from agricultural fields and banana plantations surrounding DARU (Figure 1). Indeed, runoff water sampled during one rain event (February 12, 2015) had high concentrations of inorganic nutrients (270–390, 90–140, and 1,200–5,600 μg L^–1^ of ammonia, nitrite and nitrate, respectively). These concentrations are within the range of the highest concentrations measured in the reservoir over the time-series.

Throughout the 3 years of the study period, chlorophyll *a* (chl-*a*) concentrations were in the range of ∼0.01–0.06 μg L^–1^, and were associated with high concentrations of fucoxanthin, suggesting diatoms were the dominant phytoplankton. One clear exception was during September 2013, when chl-*a* concentrations peaked (0.13 μg L^–1^), and were associated with high zeaxanthin, suggesting a cyanobacterial bloom.

### Water Quality Parameters, Fish Biomass and Feeding Differed Between the Two Fishponds

Similar to the reservoir, the temperature changed seasonally in the two fishponds, D1 and V2, and N concentrations were typically higher during the winter months (even when fish had not been stocked yet; Supplementary Figure 1). However, there were major differences between the fishponds, and between them and the reservoir. The reservoir waters consisted of significantly higher concentrations of *N* (2378 ± 266 μg L^–1^, *n* = 28; Kruskal–Wallis test, Chi square = 26.8, df = 2, *p* < 0.001; *post hoc* Wilcoxon signed-rank test, adjusted *p* < 0.001), compared to similar concentrations in the fishponds D1 (828 ± 341 μg L^–1^, *n* = 17) and V2 (589 ± 242 μg L^–1^, *n* = 18; *post hoc* Wilcoxon signed-rank test, adjusted *p* > 0.05). On the other hand, both fishponds reached up to tenfold higher concentrations of P (D1 – 59 ± 25 μg L^–1^, *n* = 17; V2 – 91 ± 21 μg L^–1^, *n* = 19), compared to the reservoir (9 ± 1 μg L^–1^, *n* = 22; Kruskal–Wallis test, Chi square = 17.66, df = 1, *p* < 0.001; *post hoc* Wilcoxon signed-rank test, adjusted *p* < 0.001). The ratio of N to P ([NH_4_ + NO_2_ + NO_3_]/SRP, which we refer to here as the *N*:*P* ratio) also strongly differed between the ponds. In both the reservoir and the fishpond D1, in half of the time points the N:P ratio was equal or above the Redfield ratio of 16N:1P (ca. 275 and ca. 16, respectively). In contrast, in fishpond V2 the median *N*:*P* ratio was close to 1. This similarity pattern between the water bodies was also observed in the chl-*a* concentrations. In the reservoir and in fishpond D1, the chl-*a* was lower (0.04 ± 0.01 mg L^–1^; and 0.03 ± 0.01 mg L^–1^, n = 29, *n* = 16, respectively) compared to the fishpond V2 (0.12 ± 0.03 mg L^–1^).

The chl-*a* concentrations during 2013 in fishpond V2 were maximal in April and September, reaching values ∼2–4-fold higher than the maximal values in the reservoir. High fucoxanthin concentrations were observed in April and high zeaxanthin concentrations in September, suggesting blooms of diatoms and cyanobacteria, respectively. In the same fishpond, during 2014 chl-*a* concentrations were maximal in May, and again were associated with fucoxanthin and hence diatoms. In fishpond V2 during 2016, and in fishpond D1 during all 3 years, the overall concentrations of chlorophyll and accessory pigments were low.

Over the 3 years of the study, the fish biomass differed between the two fishponds, D1 and V2 (Supplementary Figure 2; see section “Materials and Methods” for the estimation of biomass and feed from routine aquaculture measurements). During the times where fish were maintained in both ponds, the total estimated fish biomass was always higher in fishpond D1 than at fishpond V2 (Supplementary Figure 2). However, the estimated feed input was similar between the two fishponds. Some fish also live in the reservoir, however, they are not actively stocked nor provided with food, and thus the biomass there was assumed to be negligible compared to the densely populated fishponds.

### Seasonal Dynamics Are Observed in Reservoir and the Fishponds Microbiome Structures

To obtain an overview of the microbial populations in the reservoir and the fishponds, we sequenced 16S rRNA gene amplicons using Illumina technology. The final dataset consisted of 1,840,160 sequences from 74 samples that were assigned to 5,236 ASVs Amplicon Sequence Variants (Supplementary Table 2). Rarefaction curves reached a plateau in all of the sampled communities (Supplementary Figure 3). An estimated asymptotic extrapolation of the curves to double the amount of sequences (i.e., theoretical deeper sequencing) showed only few additional ASVs, thus suggesting that our sequencing effort was sufficient to represent most of the bacterial diversity. Although identical extraction and amplification protocols were used, we observed a strong difference in the number of sequences per sample and the number of observed ASVs between 2013–2014 and 2015, which were sequenced separately (Supplementary Figure 3). Therefore, despite the overall similarity in community composition at high taxonomic classifications (e.g., class; Supplementary Figure 1), further statistical analyses were mainly performed on the 2013–2014 samples, with separated complementary analysis of the 2015 samples.

Overall, the bacterial communities of all three water bodies were strongly dominated by the classes Actinobacteria, Alphaproteobacteria, Bacteroidia, Gammaproteobacteria, and a prominent bloom of Cyanobacteriia was observed during the summer months (Figure 2 and Supplementary Figure 1). Alpha diversity indices (Chao1, Shannon diversity index and Pielou’s evenness index) did not show any significant differences among the water bodies and did not reveal any significant changes between the seasons (Supplementary Figure 4 and Supplementary Table 2 tab “’Alpha_diversity”; Kruskal–Wallis test, *p* > 0.05). The communities of the reservoir and the fishponds shared 497 ASVs (one-fifth of total observed ASVs; Supplementary Figure 5), that comprised ca. 30–80% of the sequences in the bacterial communities. Each water body contained an additional ca. 500 unique ASVs (one third of the ASVs observed in each water body), which constituted up to 15% of the total ASVs. The rest of the ASVs were shared between two of the three water bodies.

We had expected to observe major differences between the fishponds, where high fish biomass is grown, and the reservoir, in which fish are not actively stocked. Indeed, significant differences in the bacterial community composition of the two water bodies were revealed (Supplementary Figure 6; PERMANOVA test, *F*_2_,_42_ = 1.55, *R*^2^ = 0.06, *p* < 0.01), however, seasonal effects on the microbiome appeared to be a stronger explanatory variable (PERMANOVA test, *F*_1_,_42_ = 5.5, *R*^2^ = 0.10, *p* < 0.001). This suggests similar seasonal dynamics of the microbial population in all three water bodies. Furthermore, a separate analysis of the bacterial communities in 2015 revealed similar pattern of a significant dissimilarity between the water bodies (PERMANOVA test, *F*_2_,_20_ = 1.66, *R*^2^ = 0.12, *p* < 0.01), which was lower than between the seasons (PERMANOVA test, *F*_2_,_20_ = 2.45, *R*^2^ = 0.09, *p* < 0.001).

### Specific ASVs Enriched in Each Water Body

**FIGURE 3:**
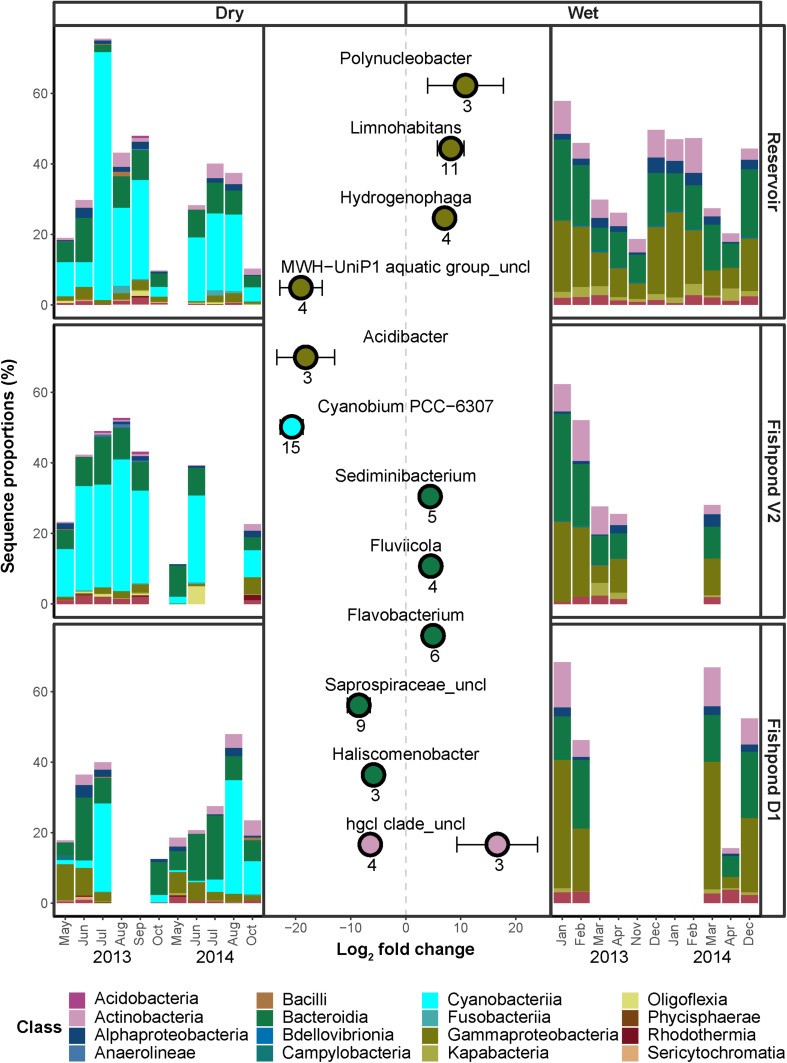
Differential enrichment analysis comparing ASV sequence abundances during dry season (Left) and wet season (Right). The *x*-axis of the central plot represents the log_2_ fold change for all significantly enriched ASVs within each labeled genera. Positive and negative values represent enrichment during wet and dry seasons, respectively. Each point and bars represent the mean and the standard deviation of the ASVs log_2_ fold change in each genera. Only taxonomic families with at least two significantly enriched (adjusted *p* < 0.1) ASVs, and an absolute log_2_ fold change value of 1, were included in the figure. The peripheral barplots represent the respective sequence proportions of the enriched ASVs within the bacterial communities of the different pools.

Using comparative sequence enrichment tests, we identified the bacterial genera that had significantly different relative sequence abundance between wet and dry seasons in all water bodies combined. We found that 84 and 74 ASVs were significantly enriched in the bacterial communities of dry and wet seasons, respectively (Figure 3). In both seasons the sequences of these enriched ASVs comprised 20–60% of the bacterial communities (Figure 3). In the wet season, the enriched ASVs of the genera *Limnohabitans* (class Gammaproteobacteria; total of 11 ASVs) comprised 8 ± 1% of the bacterial communities. In the dry season, a quarter of the enriched ASV (total of 23) were of Cyanobacteriia. Enriched ASVs of the cyanobacterial genera *Cyanobium* (15 ASVs) comprised 8 ± 1% of the bacterial communities. Furthermore, there were 2 significantly enriched ASVs from the genus *Microcystis*, which comprised 5 ± 2% of the bacterial communities. Thus, the cyanobacterial blooms are a pervasive phenomenon underlying the differences in community composition between the wet and dry seasons.

The cyanobacterial blooms were associated with the presence of cyanobacterial toxins in the water (microcystins or nodularins, which cannot be differentiated by the measuring kit used, Figure 2 and Supplementary Figure 1). To obtain a better insight into the organisms that might be producing these toxins, we focused on the dynamics of specific cyanobacterial families over time in the three water bodies (Supplementary Figure 7). Four main families contributed to these blooms: *Microcystaceae* (genus *Microcystis*), *Phormidiaceae* (genus *Planktothrix*), *Oscillatoriaceae* (an ASV related to *Planktothricoides raciborskii*) and *Cyanobiaceae* (genus *Cyanobium*; Supplementary Figure 7). *Microcystis* are well-known as producers of cyanotoxins (Wilhelm et al., 2020), with the production of similar toxins also reported for *Planktothrix* (Christiansen et al., 2003). Notably, some cyanobacterial ASVs seemed to be highly specific, appearing only ephemerally, for example an ASV related to *Sphaerospermopsis torques-reginae*, which was present at high relative sequence abundance in fishpond V2 during August 2015, but was almost completely absent at other times. There appeared to be consistent differences between the ponds in the composition of the cyanobacterial community. For example, while the reservoir and fishpond V2 were often characterized by high relative sequence abundances of *Cyanobium*, this clade was much less common in fishpond D1, which was usually dominated by *Microcystis*.

### The Effect of Physicochemical Conditions on the Bacterial Community Dynamics

**FIGURE 4:**
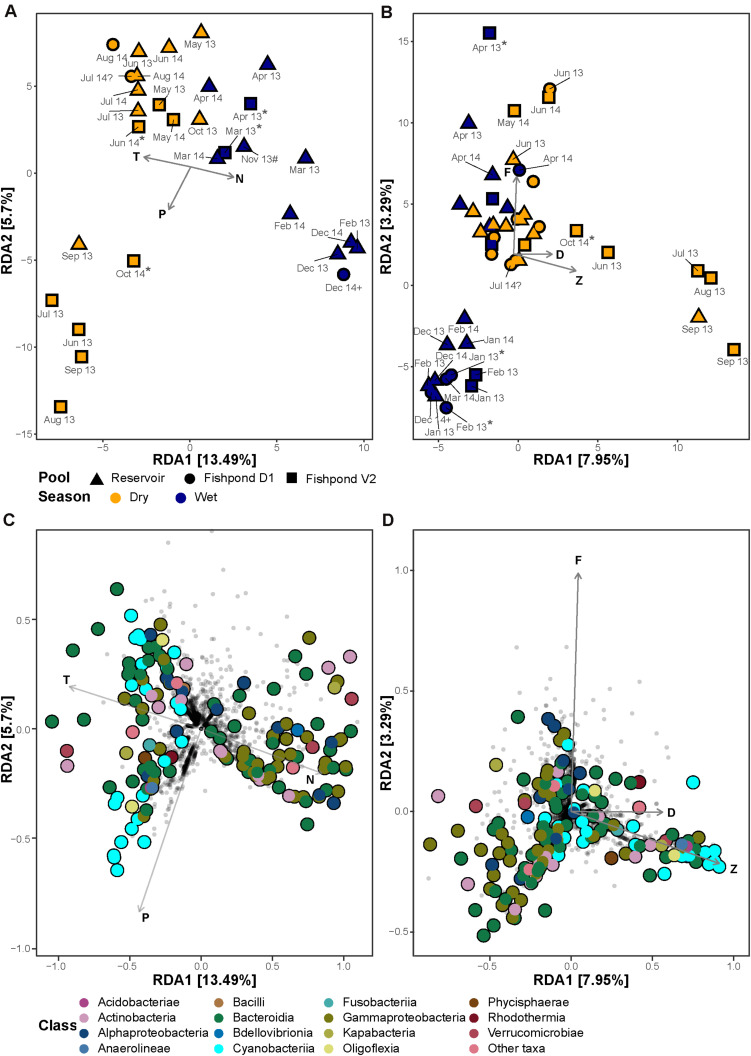
Redundancy analyses (RDA) ordination of bacterial community composition and single ASVs constrained by physicochemical parameters **(A)** and photosynthetic pigments **(B)**. Colors represent the seasons (dry – April to October; Wet –November to March) and shapes represent the different ponds. **(C)** and photosynthetic pigments **(D)**. The filled points represent significantly enriched ASVs, colored according to their taxonomic class. The percentage on the *X*- and the *Y*-axis represent the proportion of explained variance. The environmental variables are: T, temperature; N, inorganic nitrogen; P, inorganic phosphate; A, ammonia; M, microcystins; R, the N:P ratio. The pigments are: C*a*, chlorophyll *a*; C*b*, chlorophyll *b*; b, β-carotene; D, dinoxanthin; F, fucoxanthin; Z, zeaxanthin. “Unusual” samples: (*) – no fish in the fishpond, (#) – decomposed corpse of a donkey at the edge of the reservoir, (?) – fish mortality, (+) – winter puddle in a fishpond, outside of the culturing season.

In order to identify whether the observed seasonal changes in the bacterial communities are associated with natural physicochemical dynamics or with aquaculture-related dynamics we performed RDA constrained by various measured and estimated parameters. The combination of temperature, inorganic N and inorganic P concentrations showed the highest significant explanatory power (ANOVA test, *F*_1_,_26_ = 4.02, *p* < 0.001; *F*_1_,_26_ = 1.64, *p* < 0.05; *F*_1_,_26_ = 2.08, *p* < 0.01, respectively), explaining 14% of the total variation in the bacterial communities most of which along the RDA1 axis (Figure 4). These parameters separated the bacterial communities of wet and dry seasons, in accordance with the unconstrained dissimilarity analysis (Supplementary Figure 6). Temperature and N concentration showed strong negative correlation (*R*^2^ = −0.66, *p* < 0.05; Supplementary Figure 8), and separated the bacterial communities of the wet and the dry seasons. Interestingly, in 2013 we observed association between the bacterial communities of the dry season in fishpond V2 with higher P concentrations. The concentrations of P in all sampled water bodies strongly correlated to concentrations of chlorophyll *a* and other photosynthetic pigments (Pearson’s correlation; *R*^2^ = 0.48–0.58, *p* < 0.05; Supplementary Figure 8), and thus may represent distinct phytoplankton bloom conditions. An RDA constrained by concentrations of photosynthetic pigments explained 6% of the total bacterial community variation, and revealed a similar separation pattern between wet and dry seasons (Figure 4). The pigments fucoxanthin, dinoxanthin, and zeaxanthin revealed significant effect on the bacterial communities (ANOVA test, *F*_1_,_43_ = 1.58, *p* < 0.05; *F*_1_,_43_ = 1.43, *p* < 0.05; *F*_1_,_43_ = 3.14, *p* < 0.001, respectively). The dry season bacterial communities of fishpond V2 corresponded with higher concentrations of the cyanobacteria-related pigment zeaxanthin. Furthermore, aquaculture-related estimated parameters, total fish biomass and fish-feed weight, explained only 2% of the total variation in the bacterial communities and did not reveal a clear separation between the fishponds and the untreated reservoir in an RDA ordination (Supplementary Figure 9).

In addition to the effect of environmental parameters on the total bacterial community structure, a strong association was identified between specific ASVs and environmental parameters. Two clusters of ASVs associated with Bacteroidia, Gammaproteobacteria, and Cyanobacteriia were identified. In Bacteroidia, Gammaproteobacteria no clear taxonomic patterns were observed. On the other hand the cyanobacterial ASVs seemed to be associated with higher P concentrations, and were mostly associated with the species *Cyanobium gracile* (strain PCC-6307; 15/23 ASVs; Figure 4). As expected, the Cyanobacteriia enriched ASVs corresponded to higher concentrations of cyanobacterial pigment zeaxanthin (Figure 4).

## Discussion

Aquaculture facilities are anthropogenically altered hypertrophic ecosystems. Densely stocked aquaculture fishponds are supplemented with kilograms of fish-feed daily, which in most cases is not fully consumed. The remaining fish-feed, combined with fish excretions, leads to an increase in concentrations of nutrients in the water (Gowen, 1994; Talbot and Hole, 1994; Ahmed and Thompson, 2019). In addition, the fishponds often receive various pharmaceuticals (Bricknell and Dalmo, 2005; Sommerset et al., 2005; Gudding and Van Muiswinkel, 2013) including antibiotics (Lalumera et al., 2004; Cháfer-Pericás et al., 2010; Kirchhelle, 2018). The resulting conditions, which are often hypereutrophic, are very different from natural freshwater environments (Klippel et al., 2020). At our study site, we examined two intensive aquaculture fishponds that are connected to an operational reservoir, which does not contain fish biomass and is not subjected directly to the intense aquaculture practices. At the initiation of this study, we expected the water quality in the fishponds, as well as the fishpond microbiomes, to differ significantly from those in the reservoir. This is because, although connected, the water from the fish ponds is expected to undergo a process of “biofiltration” by the plants lining the connecting canals, and because the reservoir also receives input from a groundwater well. Indeed, the nutrient concentrations in the fishponds were different from those in the reservoir, characterized by higher P and lower N concentrations, leading to differences in the N:P ratio. Therefore, it was surprising that the dynamics of the bacterial communities of the three water bodies were more strongly associated with seasonal variation than with the specific water body. Similar dynamics were observed recently in the Lake Taihu, where seasonality was shown to “overwhelm” net-pens aquaculture activity (Zeng et al., 2019). Our results extend this observation to a system where the water bodies are separate (linked by limited water flow), and differences in water quality (nutrients, chl-*a*) between the water bodies are much more pronounced. In the sections below we discuss specific aspects of the DARU system in relation to seasonal and aquaculture-related variables, highlighting several caveats of our study and identifying research directions which may contribute to understanding the dynamics of microbial ecosystems in aquaculture and other anthropogenically-impacted systems.

### Temperature and Rainwater Runoff as Potential Seasonal Drivers of Water Quality and Bacterial Diversity

What are the potential environmental drivers of the observed seasonal patterns? We identified temperature as a significant explanatory variable of bacterial community composition, and indeed several studies have highlighted the importance of temperature as a driver of microbiome composition in aquatic environments (McFall-Ngai et al., 2013; Sunagawa et al., 2015). This includes aquaculture facilities in Lake Taihu as well as a semi-intensive aquaculture systems for growing sea bass and sea bream in Ria de Aveiro estuarine lagoon, Portugal (Duarte et al., 2019; Zeng et al., 2019). Temperature can also impact the gut microbiomes of fish and other aquatic organisms, such as tadpoles, which could also affect the structure of the fishpond microbiome (Kohl and Yahn, 2016; Kokou et al., 2018).

An additional seasonal factor that could affect water quality and subsequently the aquatic microbiome, is rainfall. During significant rain events, DARU receives runoff from the surrounding agricultural fields, carrying sediments, nutrients, fertilizers, pesticides and other soil-based compounds (Maltby et al., 1995; Page et al., 1995; Mallin et al., 2009; Kraemer et al., 2020; Marmen et al., 2020). Unfortunately, we currently lack a quantitative budget of the water and nutrient inputs into the reservoir and fishponds, which is needed to test the hypothesis that rainwater runoff may be important at DARU. Importantly, rainwater collected through runoff is often used as a source of water for aquaculture, and the availability of such water is likely to be reduced due to climate change (Ahmed et al., 2018). Our results highlight the need for a detailed and quantitative understanding of rainwater-driven links between aquaculture facilities and their surrounding terrestrial environments, through input of microorganisms, nutrients and potential contaminants (e.g., fertilizers and pesticides).

There were several samples which were “unusual,” including cases where the fishponds were mostly empty save for a small winter puddle at the deepest point, or when the fishponds were full of water but not yet stocked with fish. Additionally, a case of fish mortality was recorded in pond D1 during July 2014, and the carcass of a dead donkey was found in the reservoir, not far from the sampling point, during November 2013 (Supplementary Figure 1). There were no observations of pathogens or saprophytic bacteria during these events, and overall the bacterial diversity in the “unusual” samples did not reveal major differences to other samples (Figure 4 and Supplementary Figure 6). However, because these were unique occurrences no statistical test could be performed. Taken together, these observations highlight the resilience of the aquatic microbiome to “point perturbations,” and further support the hypothesis that seasonal patterns, and to a lesser extent aquaculture practices, drive the aquatic microbial population structure.

### Aquaculture Practices May Affect the Bacterial Diversity via Nutrient Concentrations and N:P Ratios

Despite the importance of seasonality in determining the microbial population at DARU, our results also revealed differences in the water quality and bacterial community composition between the two fishponds, and between them and the reservoir. The constantly full reservoir showed much higher concentrations of N, compared to the fishponds that are emptied annually and are refilled mostly with new underground water. It is therefore possible that seasonality, for example in rainwater runoff, exerts a stronger influence on the “older” waters of the reservoir. On the other hand, both fishponds revealed tenfold higher SRP concentrations, compared to the reservoir. The much higher concentrations of P in the fishponds are likely a result of the nutritional input into them, as the fish-feed used had an estimated 8% N (50% protein times 16% N in protein) compared to 1.5% PO_4_, resulting in an N:P ratio of 5.3:1, compared to the Redfield ratio of 16:1. Indeed, the concentration of SRP in the water increased during the fish-stocking season.

We propose that the differences in the timing, amplitude, and potential source of nutrient input into the system led to pronounced differences in the N:P ratio between the three water bodies. The ratio between inorganic N and P is often used to infer the limiting nutrient for phytoplankton growth (Deutsch and Weber, 2012). Based on this logic, the very high N:P ratios in the reservoir suggest that primary production of this freshwater body is limited by availability of P. Fishpond D1 was likely also P-limited, whereas fishpond V2 was likely mostly N limited. The differences in nutrient concentrations, and potentially in the identity of the limiting nutrient, may be due to the fact that, while the input of fish-food was similar between the two ponds, the fish biomass was typically several-fold higher in pond D1 (Supplementary Figure 2). This discrepancy between food input and the expected utilization by the fish was especially pronounced in the seasons of 2013. Notably, chl-*a* concentrations were typically higher in fishpond V2 compared to D1 and the reservoir. It is tempting to speculate that the higher chl-*a* concentrations in fishpond V2 are a result of the surplus P in this water body, although other causes such as differences in zooplankton or fish grazing cannot be ruled out. The bacterial communities of fishpond V2 during the dry season of 2013, where chl-*a* was high, also showed a strong association with higher concentrations of P. Therefore, we hypothesize that characteristics (e.g., magnitude) of the phytoplankton bloom, including also cyanobacteria, may be influenced by aquaculture-associated processes, in turn affecting the microbial communities. As noted above, a quantitative nutrient budget for aquaculture facilities is required to test this hypothesis, taking into account both environmental sources of nutrients (e.g., rainwater runoff) and aquaculture related sources and sinks (fish food, growth yield, and excretion). Such a quantitative budget should also take into account the connectivity between the water bodies and the flow of microorganisms, in order to test to what extent microbial populations are linked, and to determine the relative contribution of selection within each water body (as proposed above) compared with neutral processes such as emigration and founder effects (e.g., Langenheder and Székely, 2011).

### Cyanobacterial Blooms as Major Seasonal Occurrences in Aquaculture

Perhaps the most clearly observed seasonal change in the bacterial community structure were the summer cyanobacterial blooms. As a group, cyanobacteria generally exhibit optimal growth rates at relatively high temperatures, usually in excess of 25°C (Robarts and Zohary, 1987; Paerl and Huisman, 2009) and the formation of cyanobacterial blooms is encouraged by warm and calm weather (Kanoshina et al., 2003). These conditions are prevalent during the summer months in Israel. Certain cyanobacteria can produce metabolites that cause undesirable flavors to the fish products (Paerl and Tucker, 1995; Schrader and Dennis, 2005; Mares et al., 2009; Lee et al., 2017; Lindholm-Lehto and Vielma, 2019; Tucker and Schrader, 2020), and/or various toxins that can kill both invertebrates and fish (Oberemm et al., 1999; Smith et al., 2008). Over the course of this study, cyanotoxins (microcystins or nodularins) were repeatedly observed in the particulate fraction of the reservoir and the fishponds during summer, at concentrations that, at times, were above the recommended limit for drinking water of 1 μg microcystins L^–1^ [according to guidelines set by the World Health Organization (World Health Organization, 2008)]. Microcystins have repeatedly been documented in aquaculture ponds worldwide, at concentrations similar to, or higher than, observed here (Ahmed et al., 2008; Chia et al., 2009). In a survey of 485 catfish production ponds across the Southeastern United States, microcystins were detected in 47% of the ponds, with ca. 10% of the ponds containing microcystin concentrations were above the World Health Organization limit (Zimba and Grimm, 2003). These concentrations may cause damage to fish tissues, as demonstrated in a survey of fishponds in Serbia (Drobac et al., 2016). Additionally, there is evidence that microcystins affect human epidermal skin cells (Kozdȩba et al., 2014), and that exposure to non-lethal doses (0.5 μg L^–1^) may contribute to the development of hepatocellular carcinoma (Yoshizawa et al., 1990; Yu, 1995; Pearson et al., 2010). Given that many aquaculture workers are often not aware of the potential presence of toxins in the water, future research assessing the effects of chronic exposure, followed if needed by appropriate communication and training, are important.

While microcystins or nodularins were observed in the water of the reservoir every summer, the cyanobacteria that produced the blooms differed from year to year, and even between consecutive months of the same year (Supplementary Figure 7). *Microcystis* are one of the most common bloom-forming, and potentially toxin-producing, cyanobacteria (Wilhelm et al., 2020). Blooms of *Microcystis*, including toxic species, are a well-known issue in Israel, particularly for growers of the Common carp in the northern coastal region and the Beit She’an valley (e.g., Elkobi-Peer and Carmeli, 2015). In fact, in our previous work, we detected the presence of *Microcystis* with the potential of microcystins production in all sampled fishponds in Israel (Marmen et al., 2016). Such blooms are also common elsewhere in the world (Zimba et al., 2001; Paerl and Paul, 2012; Berdalet et al., 2018). However, in this study there were times during which, based on the 16S rRNA amplicons, *Microcystis* was almost absent, yet microcystins were present in the water. For example, a major bloom of *Planktothricoides raciborskii* was observed in the reservoir during July 2013 (ca. 60% of the sequences), with relatively high microcystin concentrations (Figure 2 and Supplementary Figure 7). However, during this bloom less than 1% of the sequences were associated with *Microcystis*. At this time, it is possible that the microcystins were produced by a third organism, *Planktothrix*, that comprised ca. 8% of the sequences (Christiansen et al., 2003).

Finally, the cyanobacterial clade that differed between the wet and dry periods was *Cyanobium*. Relatively little is known about this clade of pico-cyanobacteria (Komárek et al., 1999; Pagels et al., 2020), despite its observed dominance in cyanobacterial blooms, including toxic ones (Li et al., 2020). They are typically found as free-living single cells, but can produce colonies in response to grazing pressure (Jezberová and Komárková, 2007; Huber et al., 2017). They have also been shown to produce allelopathic substances, although there is currently no evidence that they can produce microcystins or nodularins (Costa et al., 2015; Kovács et al., 2018). Multiple ASVs of this clade were observed, and more research is needed in order to determine which factors govern the dynamics of this microorganism in the DARU system or in freshwater environments in general.

### Are Heterotrophic Bacteria Associated With Cyanobacterial Blooms or Aquaculture Practices?

In this study, we identified major changes in the bacterial community structure between the wet and dry seasons, and smaller changes potentially associated with the aquaculture activity. While cyanobacteria were clearly associated with the dry season, several other genera of bacteria were enriched in the dry or wet seasons. Notably, due to the fractional (relative) nature of 16S rRNA amplicon sequencing, changes in relative sequence abundance of an ASV cannot be equated with actual changes in the cell numbers of the relevant organisms (McLaren et al., 2019). The increase in cyanobacterial sequences, and the enrichment of cyanobacterial ASVs during the dry season, are likely due to blooms of these organisms, since increases were observed in both zeaxanthin, a pigment associated with cyanobacteria (Zhang et al., 2018), and cyanotoxins. For this reason, it is unclear whether the enrichment of genera such as *Polynucleobacter*, *Limnohabitans* and *Hydrogenophaga* in the wet season represents bona-fide blooms of these organisms, or whether this is a result of the decrease in relative sequence abundance of cyanobacteria. In contrast, enrichment of ASVs in the dry season may be easier to interpret as bona-fide biological patterns in abundance. In this regard, the enrichment of nine ASVs belonging to the family *Saprospiracae* clade during the dry season is intriguing, as this clade is thought to be associated with the hydrolysis and utilization of complex carbon sources as well as with active predation on bacteria and algae, including *Microcystis* (Ashton and Robarts, 1987). Additionally, both *Saprospiracae* and another group enriched in the dry season, the genus *Haliscomenobacter*, has been suggested to be associated with the presence of both cyanobacterial blooms and antibiotic resistance genes in Lake Taihu (Zhang et al., 2020). While antibiotics are not used directly in the three sampled water bodies, they are used prophylactically at DARU at the beginning of the fish fattening cycle (beginning of dry season), including in additional fishponds connected to the system (Patil et al., 2016, 2020).

It has previously been suggested that the microorganisms in the aquaculture water may have a direct effect on the health of the fish (Kim and Lee, 2017; Assefa and Abunna, 2018). For example, environmental microorganisms can form the initial inocula for fish eggs or larvae, and pathogens can be transferred through the water, especially when fish biomass/density is very high. Therefore, significant effort has been invested in studying the aquatic microbiome in some aquaculture types, with the aim of using these measurements to understand fish health, to identify biomarkers/early markers of emerging diseases, or to explore the use of probiotics (Dawood et al., 2019; Kuebutornye et al., 2019; Kolda et al., 2020; Zhang et al., 2020). In our dataset, we identify several bacterial lineages known as fish pathogens, including the genera *Aeromonas*, *Vibrio*, and *Enterobacter*, in agreement with previous studies from DARU (Patil et al., 2016, 2020). However, due to the limited phylogenetic resolution of partially sequenced 16S rRNA gene, it is usually not possible to identify whether the observed taxa are associated specifically with pathogenic strains. For such analysis, one would need to analyze additional markers, for example genes related directly with pathogenicity. Nevertheless, we did identify one potentially interesting pattern, whereby *Vibrio*-related ASVs were found almost only in fishpond D1, and specifically during May and June of 2013 and 2014 (but not 2015), during which the *Vibrio* comprised 3–12% of the bacterial community. During these 3 years, fishpond D1 was used for Common carp outgrowth, however, no major cases of pathogen outbreaks were observed in the fishpond. Interestingly, during 2014 and 2015, fishpond V2 was used for outgrowth of Common carp larvae, but we haven’t detected *Vibrio* presence there. Thus, further study is needed to identify potential reasons for the strong association of *Vibrio* with a specific fishpond and time, and to what extent this is associated with changes in fish health.

## Conclusion

As human impact on aquatic environments increases, it is imperative to understand how microbial communities will respond to anthropogenic stressors, and to differentiate between these responses and natural ecosystem dynamics. The aquaculture unit at DARU represents a unique semi-controlled system where water bodies under strong anthropogenic pressure – densely stocked fishponds – are connected with a water body (the reservoir) which is not directly subject to these intense pressures. Our results suggest that, even in this highly modified aquatic ecosystem, natural seasonality (e.g., temperature and rainwater runoff) is the main driver of the bacterial diversity. Perhaps the clearest manifestation of the natural cycle is the presence of cyanobacterial blooms during summer. However, the cyanobacterial bloom dynamics, which at times also included toxin-producing organisms, seem to be affected by aquaculture practices, such as fish feeding. Thus, based on the results presented here, we suggest that nutrient dynamics and possibly elemental ratios in freshwater environments, including in aquaculture, should be taken into account in the management of such ecosystems. Resolving such dynamics requires quantitative budgets of the flow of water, nutrients and microorganisms within the system and between it and its environment.

## Data Availability Statement

The datasets generated for this study can be found in online repositories. The names of the repository/repositories and accession number(s) can be found in the article/Supplementary Material.

## Author Contributions

SM: conceptualization, data curation, investigation, formal analysis, visualization, writing – original draft, and review and editing. EF: data curation, formal analysis, visualization, writing – original draft, and review and editing. AA: data curation, formal analysis, visualization, writing – original draft, and review and editing. AB-P: conceptualization, investigation, and writing – review and editing. AN, HP, and YV-M: investigation and writing – review and editing. EC and AS: cnceptualization, funding acquisition, and writing – review and editing. ML: data curation, formal analysis, supervision, and writing – review and editing. DS: conceptualization, project administration, investigation, formal analysis, visualization, writing – review and editing, supervision, and funding acquisition. All authors contributed to the article and approved the submitted version.

## Conflict of Interest

The authors declare that the research was conducted in the absence of any commercial or financial relationships that could be construed as a potential conflict of interest.

## Footnotes

1 http://www.Rproject.org/

2 http://www.rstudio.com/

3 https://benjjneb.github.io/dada2/tutorial.html
