# Supplementary material for: Seasonal Dynamics Are the Major Driver of Microbial Diversity and Composition in Intensive Freshwater Aquaculture
Source: Front Microbiol. 2021 Jun 24;12:679743. doi: 10.3389/fmicb.2021.679743 (PMC8264503; doi:10.3389/fmicb.2021.679743)
Supplement: Supplementary file 2 [file Data_Sheet_1.pdf]

Supplementary material

Seasonal dynamics are the major driver of microbial diversity and composition in intensive freshwater aquaculture

Sophi Marmen[1†], Eduard Fadeev[2†], Ashraf Al Ashhab[1,3], Ayana Benet-Perelberg[4], Alon Naor[4], Hemant J. Patil[5], Eddie Cytryn[5], Diti Viner-Mozzini[6], Assaf Sukenik[6], Maya Lalzar[7] and Daniel Sher[1]*

[1]*Department of Marine Biology, Leon H. Charney School of Marine Sciences, University of Haifa, Haifa, Israel*

[2]*Department of Functional and Evolutionary Ecology, University of Vienna, Vienna, Austria*

[3]*Microbial Metagenomics Division, The Dead Sea and Arava Science Center, Masada 86900, Israel*

[4]*Dor Aquaculture Research Station, Fisheries Department, Israel Ministry of Agriculture and Rural Development, Israel*

[5]*Institute of Soil, Water and Environmental Sciences, Volcani Center, Agricultural Research Organization, P.O Box 15159, Rishon Lezion, 7528809, Israel*

[6]*The Yigal Allon Kinneret Limnological Laboratory, Israel Oceanographic and Limnological Research, P.O.Box 447 Migdal 14950, Israel*

[7]*Bioinformatics Service Unit, University of Haifa, Israel*

†SM and EF contributed equally to this study

***Correspondence:**

Eduard Fadeev - eduard.fadeev@univie.ac.at

Figures

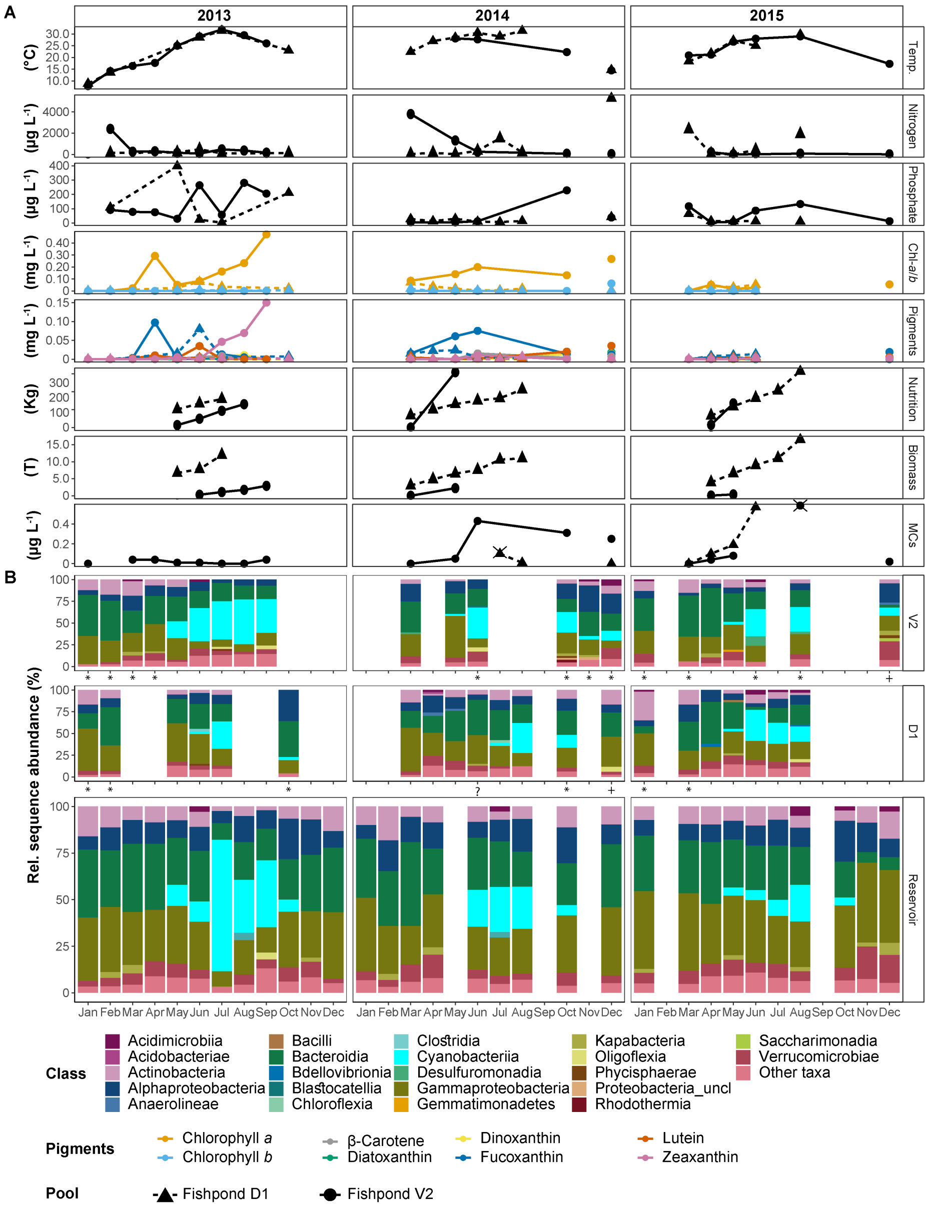

Figure S1. Seasonal dynamics in the fishponds between 2013 and 2015. (A) Monthly measured physicochemical properties of the water. The different photosynthetic pigments are colored according to the legend. Shapes represent the different fishponds. Nitrogen represents the total concentration of nitrate and nitrite. In microcystins (MCs), samples marked with '×' represent potentially underestimated concentration. (B) Sequence proportion overview of bacterial communities on a class level in the fishponds and the reservoir. The classes represented by colors according to the legend, all classes with sequence proportions below 2% were classified as "Other classes". Special conditions in fishponds: (*) - no fish in the fishpond, (?) - fish mortality, (+) - winter puddle in a fishpond, outside of the culturing season.

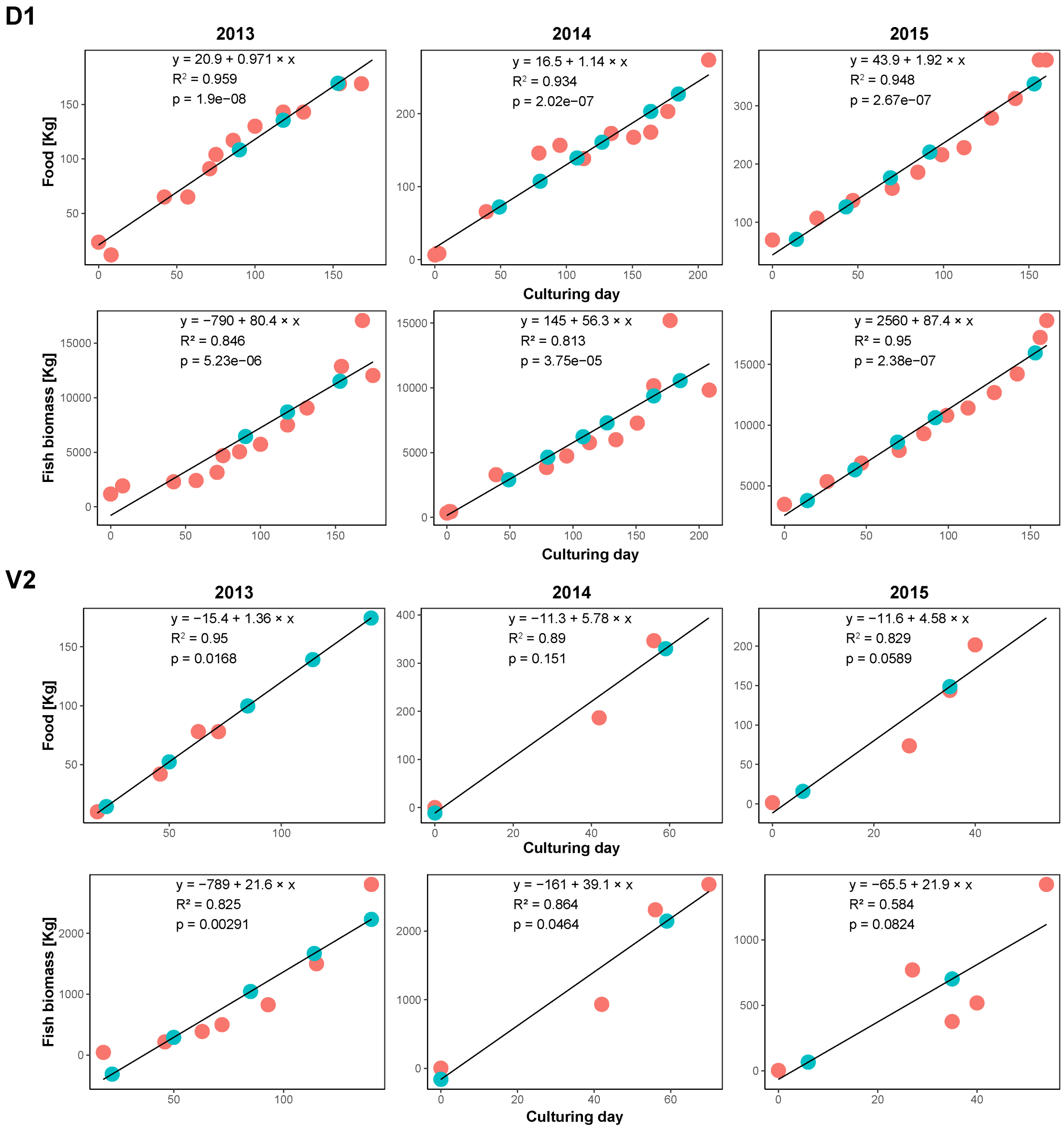

Figure S2. Linear regression models of nutrition input and fish biomass during the culturing seasons of 2013-2015 in the fishponds D1 and V2. The red dots represent measured values that were used for building the models, and the blue dots represent the predicted values in time points where microbial sampling was not complemented with aquaculture measurements. Each panel represents the independent model and its significance.

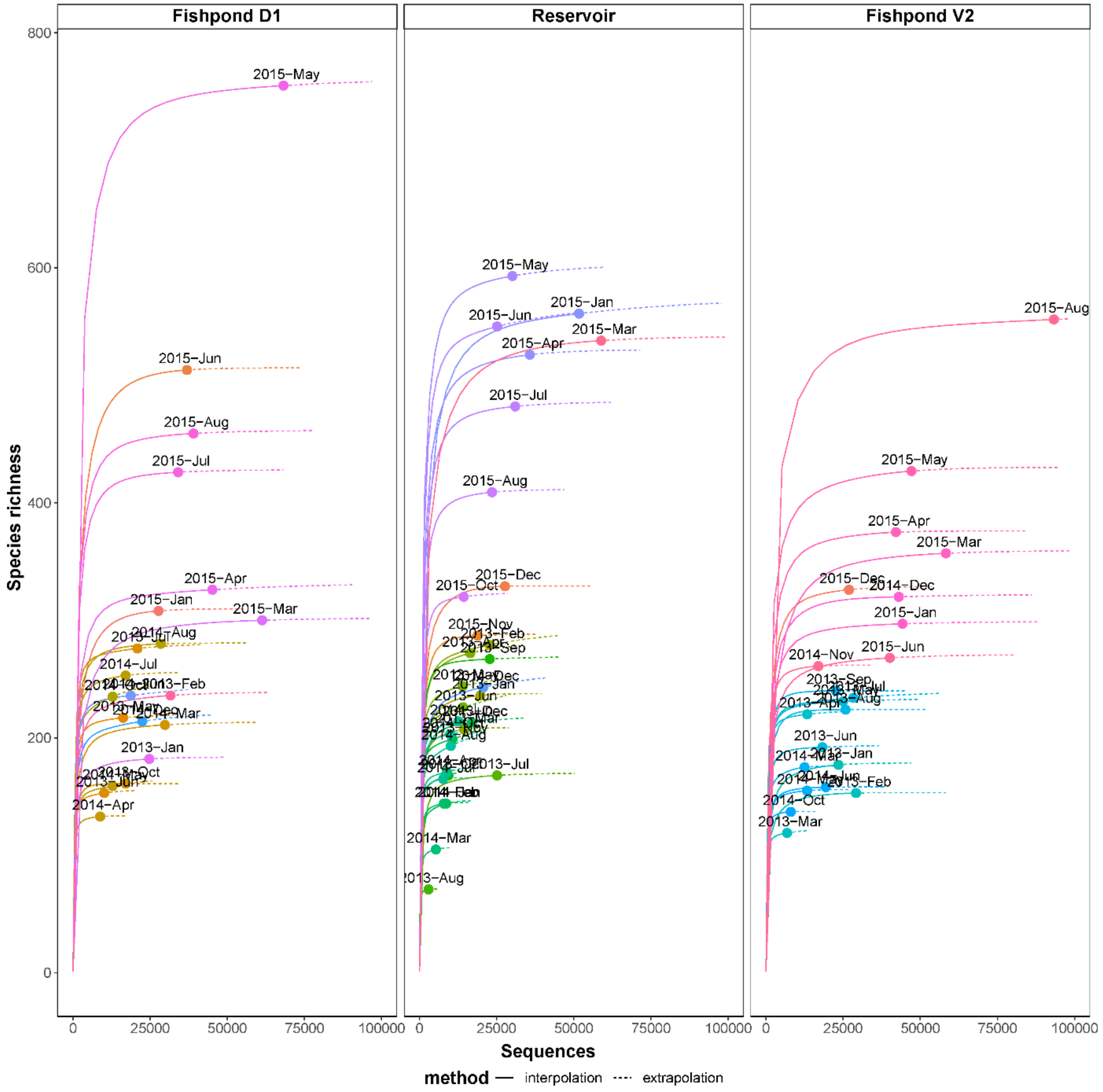

Figure S3. Rarefactions of 16S rRNA gene analysis of bacterial communities in the reservoir and the fishponds. The solid lines represent the observed accumulation with the number of reads sampled, and the dashed lines represent the extrapolated accumulation up to the double amount of reads. The observed values for each community are denoted by solid shapes. Sample-size-based rarefaction curves generated with the R-package “iNEXT”, based on the Hill number of order q = 0. The rarefaction curves for each sample were generated based on 40 equally spaced rarefied sample sizes with 100 iterations.

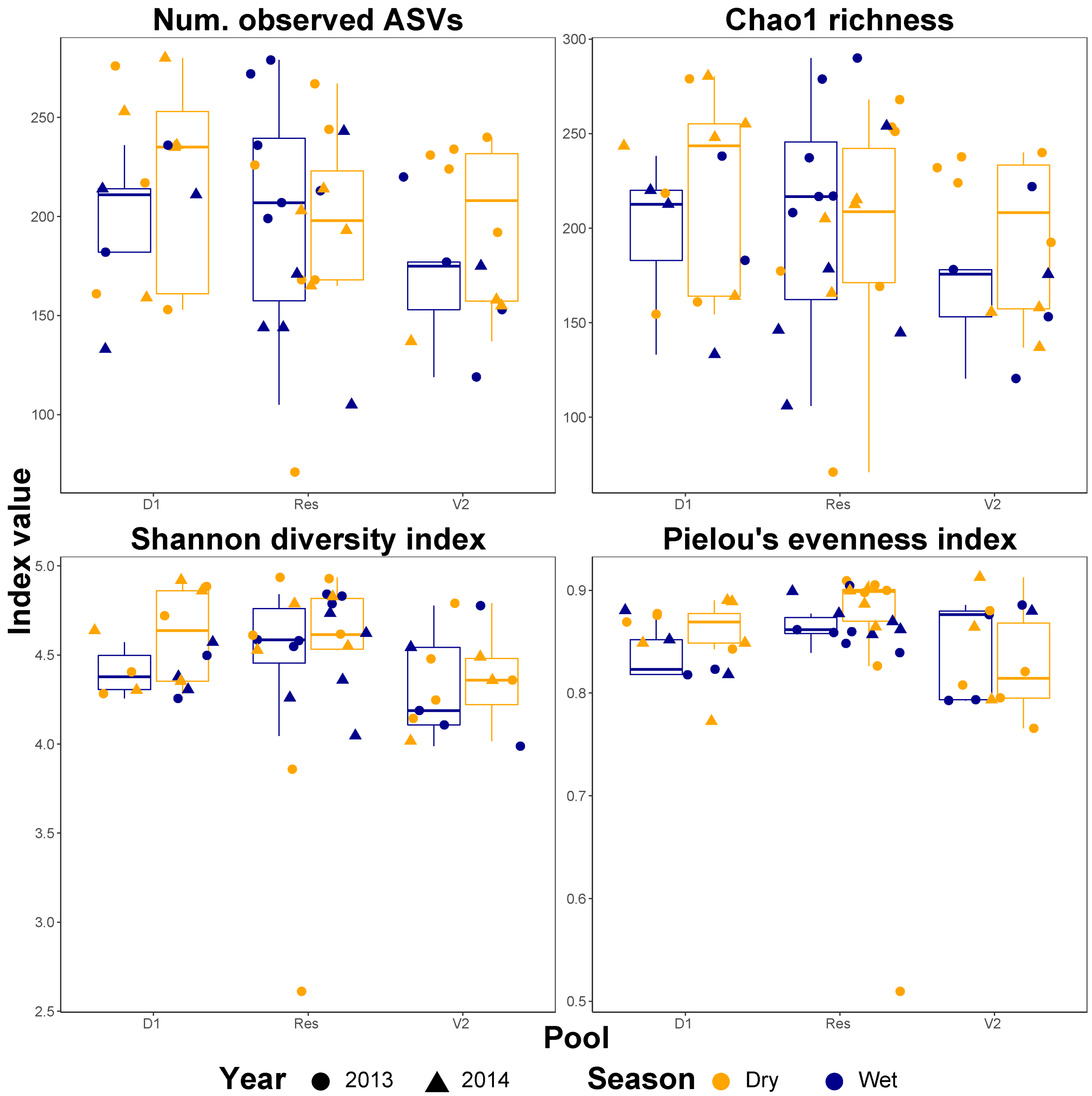

Figure S4. Alpha diversity indices of the bacterial communities in the reservoir and the fishponds. The shapes represent years and the colours the sampled season, according to the legend.

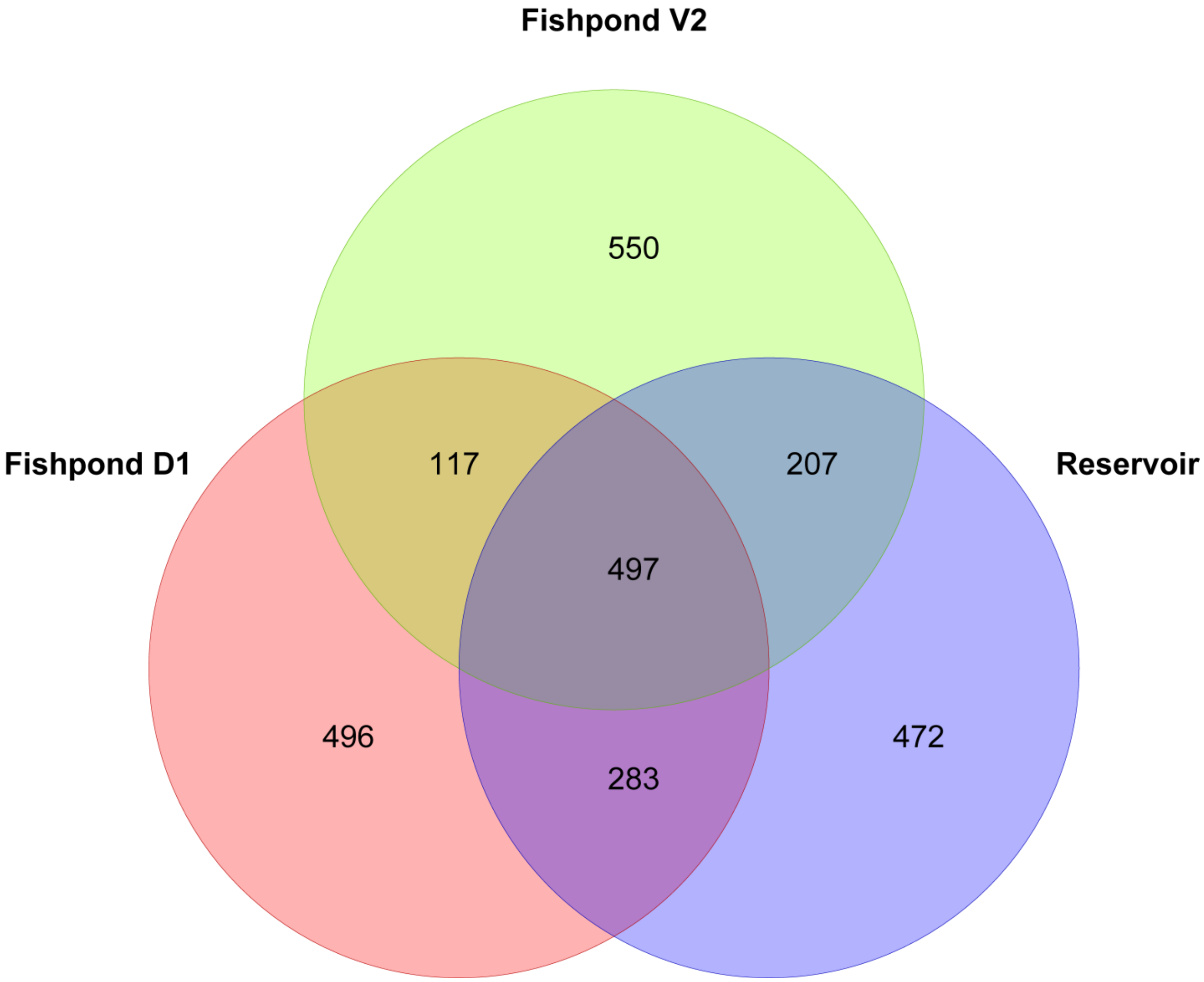

Figure S5. Venn diagram of shared and unique ASVs between the bacterial communities of the reservoir and the fishponds.

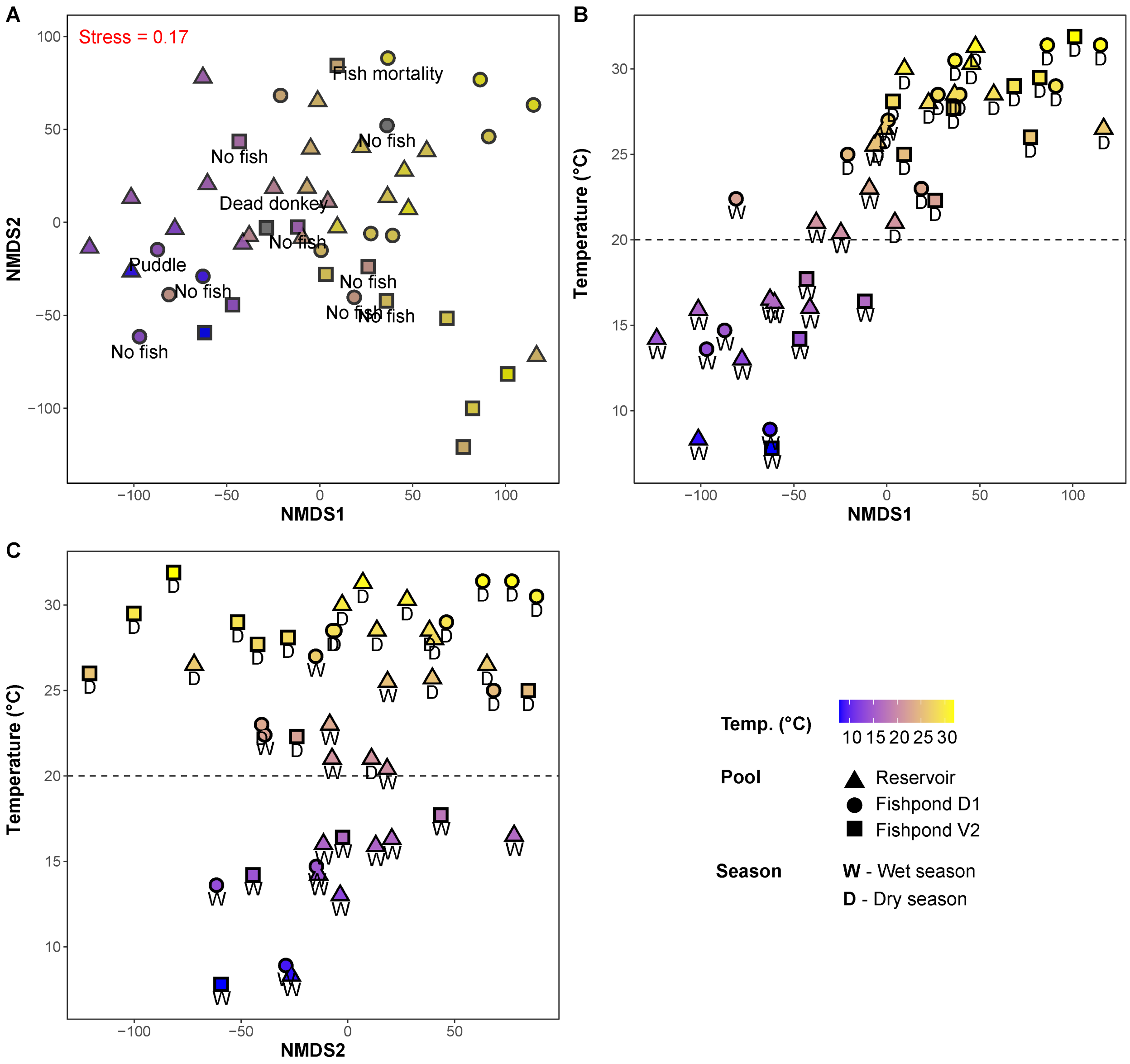

Figure S6. Non-metric multidimensional scaling ordination bacterial communities of the reservoir and the fishponds. The color range represents the water temperature of each sampling time point. Shapes represent the different water bodies, and letters represent the seasons, according to the legend. In panel a the labels describe special conditions with potential ecological impact. “No fish” – no fish were present in the pond at the time of sampling. “Dead donkey” – a corpse of a donkey at a stage of advanced decomposition was observed at the edge of the reservoir (donkeys were maintained at DARU to assist in minimizing plant growth around the ponds). “Puddle” - water remaining in the deepest part of the fishponds, outside of the aquaculture season.

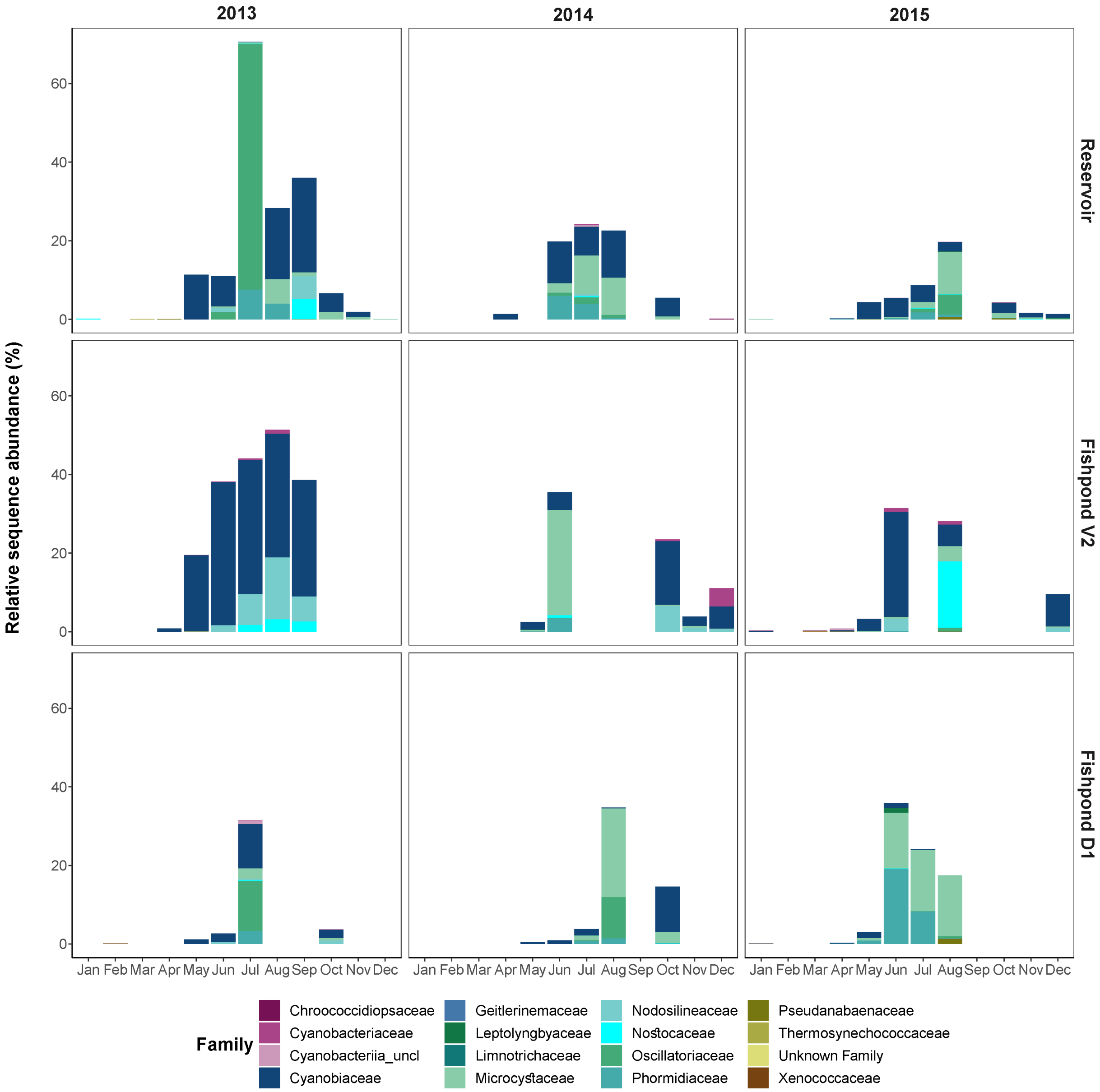

Figure S7. A comparison of the fraction of reads belonging to cyanobacterial families in the reservoir and the fishponds.

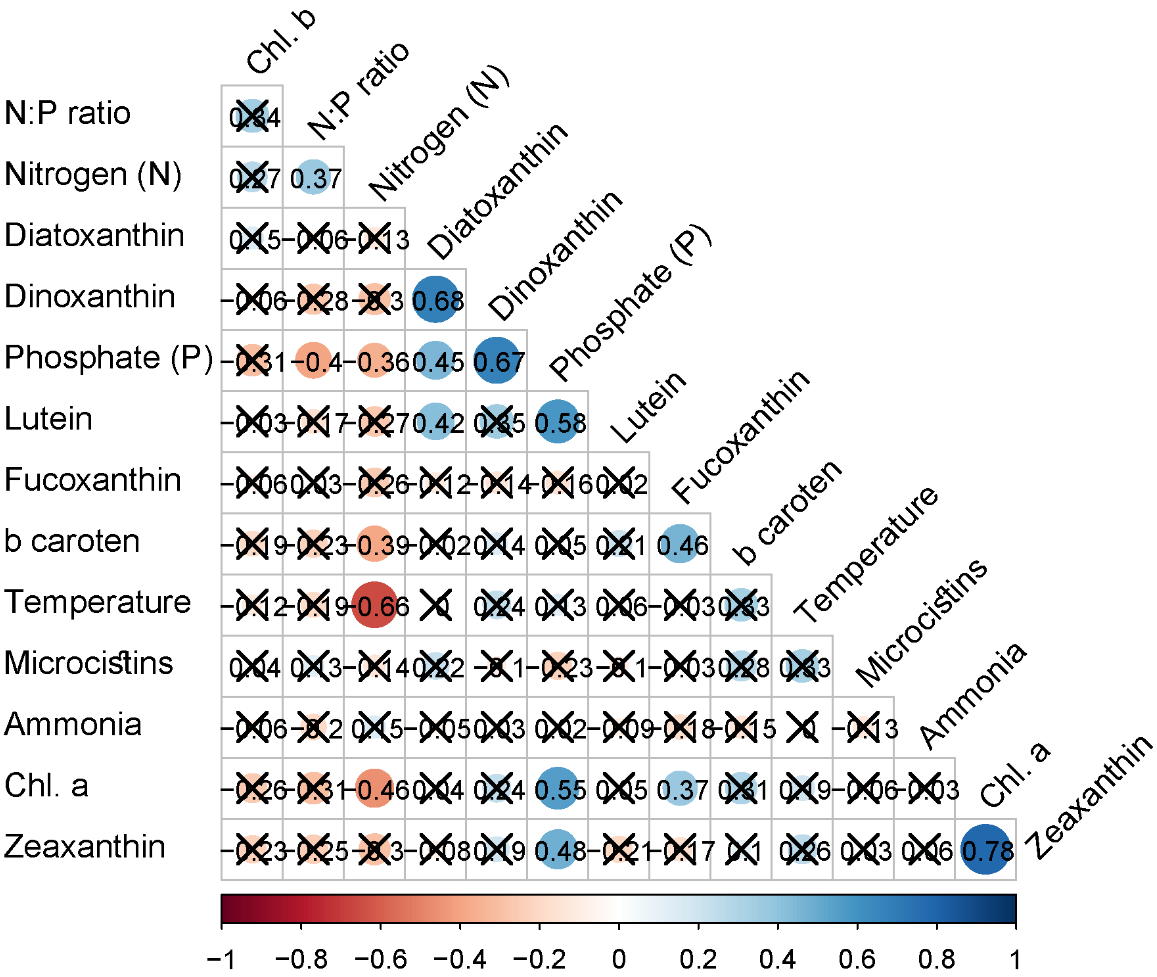

Figure S8. Pearsons' correlations between measured and estimated environmental parameters. The non-crossed out values represent significant correlations. Nitrogen represents the total concentration of nitrate and nitrite. The correlation strength is represented by color according to the legend. The order of the parameters is defined by hierarchical clustering of their correlations.

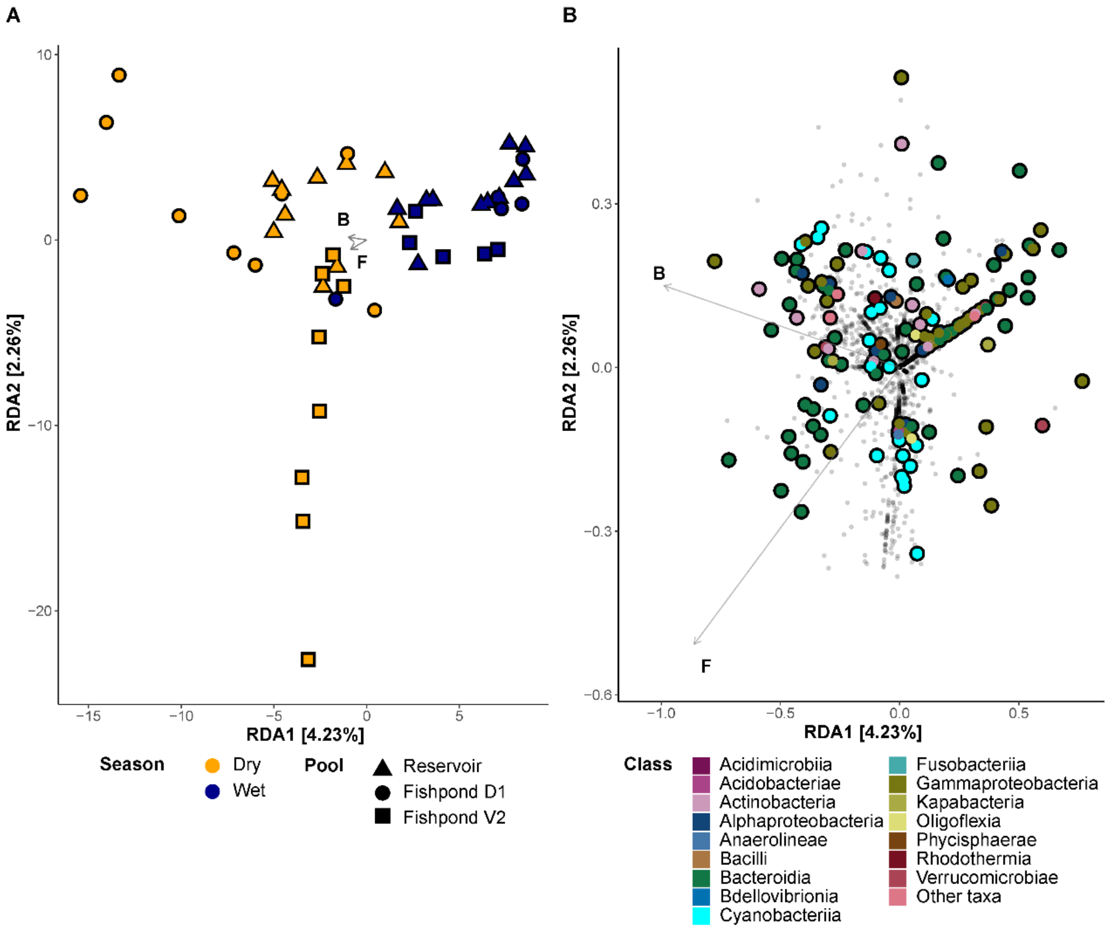

Figure S9. RDA ordination of bacterial community composition constrained by aquaculture-related variables. (a) Communities constrained ordination. Colors represent the seasons and shapes represent the different pools. (b) Bacterial ASVs constrained ordination. The large points represent enriched ASVs, colored according to their taxonomic class. The environmental variables are: F - feeding input, B - total fish estimated biomass.

Tables

Table S1: Fish culturing duration and sampling time intervals at different fishponds.

**Year**	**Fishpond**	**Fish Species**	**Stocking period**	**Number of sampled timepoints (n)**	**Final fish biomass [t]**	**Total food input [kg]**
2013	D1	*Hypophthalmichthys molitrix*	March-July	3	12.4	421
	V2	*Cyprinus carpio*	May-September	5	2.7	411
2014	D1	*Cyprinus carpio*	March-August	6	13.8	966
	V2	*Cyprinus carpio*	April-June	2	2.1	381
2015	D1	*Cyprinus carpio*	April-August	5	16.6	721
	V2	*Cyprinus carpio*	April-May	1	2	144
